# Forensic toxicology of benzodiazepines: neuropharmacological effects, analytical challenges, and emerging detection strategies

**DOI:** 10.3389/ftox.2025.1639890

**Published:** 2025-09-25

**Authors:** Husna Irfan Thalib, Ahmed Abdelghany Damanhory, Ayesha Hanin Shaikh, Shyma Haidar, Sariya Khan, Ayesha Jamal, Omar Ahmed Abdelghany

**Affiliations:** ^1^ General Medicine Practice Program, Batterjee Medical College, Jeddah, Saudi Arabia; ^2^ Department of Biochemistry, General Medicine Practice Program, Batterjee Medical College, Jeddah, Saudi Arabia; ^3^ Kasr Al-Ainy, Faculty of Medicine, Cairo University, Cairo, Egypt

**Keywords:** benzodiazepines (BZDS), drug-facilitated crimes (DFCS), forensic detection, cognitive impairment, legal challenges

## Abstract

The increasing misuse of benzodiazepines (BZDs) in drug-facilitated crimes (DFCs) has become a serious concern for forensic experts, healthcare professionals, and legal authorities. These drugs, which are commonly prescribed for anxiety and sleep disorders, are also used to commit crimes such as sexual assault and robbery. Their sedative and memory-blocking effects render them particularly dangerous. One of the biggest challenges is that BZDs are rapidly broken down in the body, limiting the time available for detection. This creates major problems in forensic investigations and reduces the chance of holding offenders accountable for their actions. In addition, memory loss caused by BZDs often affects a victim’s ability to recall events, making legal cases more challenging. This review aims to comprehensively synthesize the current knowledge on the use of BZDs in DFCs, their neuropharmacological mechanisms, and the challenges associated with their detection. It also discusses legal issues and emerging forensic tools that may help overcome the current limitations. By addressing this issue from medical, forensic, and legal perspectives, this review aims to recommend better prevention strategies, more effective investigations, and stronger legal outcomes for cases involving benzodiazepine-facilitated crimes.

## Introduction

Drug-facilitated crimes (DFCs), especially sexual assault, are a major global concern. These crimes involve the covert use of psychoactive substances to reduce awareness or erase memory, leaving victims vulnerable. Benzodiazepines (BZDs) are the most common agents because of their strong sedative and amnesic effects (García et al., 2021; Pérez et al., 2023).

While indispensable for treating anxiety, insomnia, seizures, and acute agitation, BZDs also carry risks of misuse and dependence. Prolonged use promotes tolerance and withdrawal, and co-ingestion with other central nervous system depressants increases toxicity. In some cases, prescribed use transitions to diversion or illicit use, bridging medical and criminal contexts. This therapeutic–misuse spectrum provides the foundation for understanding their forensic relevance.

Even low doses of BZDs can cause drowsiness, confusion, and memory loss. Combined with alcohol or other depressants, these effects intensify, making them attractive to offenders (Edinoff et al., 2021). Many incidents remain unreported due to stigma and fear, particularly among women (Mellen et al., 2024; Kang et al., 2005). Detection is also difficult: BZDs are rapidly metabolized, and standard toxicology screens often fail to capture newer designer analogues (Fernández-López et al., 2024). Legal systems face further challenges, as impaired memory complicates testimony and consent laws remain inconsistently applied (Edinoff et al., 2022).

This review summarizes the pharmacological, forensic, and legal aspects of BZD-related DFCs and outlines strategies to improve prevention, detection, and judicial outcomes.

### Mechanism of action and receptor subtype selectivity of benzodiazepines

**FIGURE 1:**
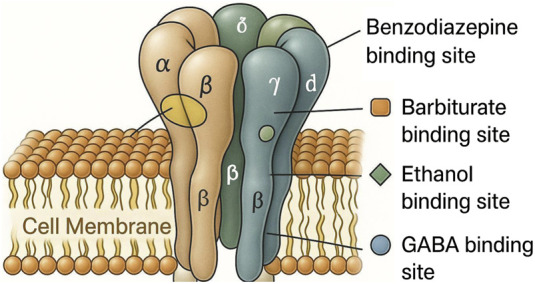
Structure of the GABA-A receptor embedded in the cell membrane, showing two alpha, two beta, and one gamma subunits. The binding sites for GABA, benzodiazepines, barbiturates, and ethanol were highlighted.

GABA (gamma-aminobutyric acid) is a neurotransmitter that has major inhibitory effects on the central nervous system of the body. GABA has two main receptor subtypes: GABAA and GABAB (Jembrek and Vlainic, 2015). GABAA is a ligand-gated chloride ion channel receptor that holds the benzodiazepine receptor subunits, which form the benzodiazepine binding site and is known as the GABAA-benzodiazepine receptor complex (Figure 1) (Jembrek and Vlainic, 2015; IIorio et al., 2020). Benzodiazepines, such as diazepam, are central nervous system depressants composed of a unique molecular structure with 6-carbon benzene rings and a 7-carbon diazepine ring with two attached nitrogen atoms. The GABAA receptor is typically composed of two α subunits, two β subunits, and one γ subunit, all of which assemble together to form the GABAA receptor complex (Nicholson et al., 2018).

**FIGURE 2:**
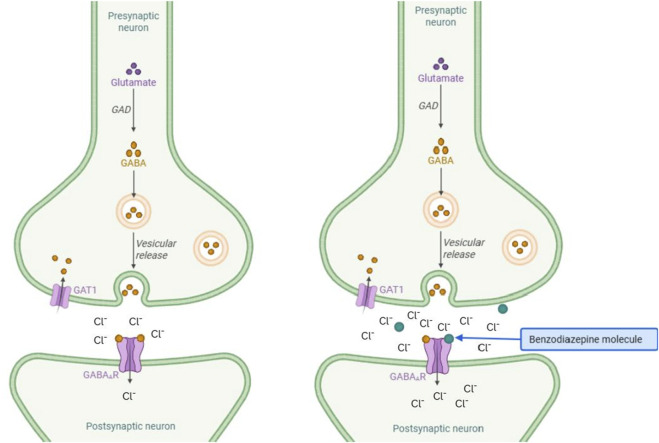
Mechanisms of action of BZD. The figure on the left represents the control condition with a moderate chloride influx. The right panel shows the increased influx of chloride ions in the presence of benzodiazepines.

After crossing the blood-brain barrier, benzodiazepines bind to their receptor-binding site located at the α/γ interface of the GABAA receptor. This binding induces a conformational change in the GABAA receptor, which enhances its affinity for GABA and results in the opening of ligand-gated chloride channels. This opening causes an influx of chloride ions, leading to the hyperpolarization of neurons (Figure 2). An increase in the influx of negative charges into the cytosol reduces the excitability of neurons controlling cognition, emotions, muscle tension, and vigilance, which explains the symptoms of CNS depression, such as moderate-to-profound sedation, dizziness, muscle weakness, anterograde amnesia (inability to form new memories), and slurred speech (Nicholson et al., 2018).

The GABAA-α1 subtype and GABAA-α2/α3/α5 subtypes have been classified as different binding site subtypes on the GABAA receptor complex, which is a single receptor composed of various subunits. The GABAA-α1 subtype is associated with the α1 subunit isoform, particularly in the cortex, thalamus, and cerebellum. It is responsible for anterograde amnesia and sedative and anticonvulsive effects. In contrast, GABAA-α2/α3/α5 subtypes are associated with the α2 isoform and are largely involved in the anxiolytic effects. They are predominantly found in the limbic system, motor neurons, and dorsal horn of the spinal cord (Griffin et al., 2013). The formerly known peripheral benzodiazepine receptor was identified using a new terminology called the Translocator Protein, 18 kDa (TSPO). This 18 kDa TSPO is specifically localized to the outer mitochondrial membrane and is also found in glial cells and peripheral tissues. It is not related to the GABA receptor, and its roles include cell proliferation, heme synthesis, calcium flow, and apoptosis (Morohaku et al., 2013).

### Mechanism of action and pharmacokinetics of benzodiazepines in drug- facilitated crimes

Although BZDs share a common mechanism of action, their pharmacokinetic and pharmacodynamic profiles vary significantly, thereby influencing their potential for DFCs misuse. Short-acting BZDs, such as midazolam (half-life: 1.5–3 h), induce rapid sedation and anterograde amnesia, making them high-risk for administration in assaults. Since midazolam is very short-acting, even a brief delay to blood/urine sampling can return false-negative results in DFSA investigations, narrowing the evidentiary window (Conway et al., 2016). In contrast, long-acting BZDs, such as diazepam (half-life: 42 h), lead to prolonged impairment of recognition memory and recollection of stories, but with less pronounced amnesia. Diazepam’s long half-life extends detectability but can obscure timing of ingestion (therapeutic vs. criminal exposure), requiring careful interpretation alongside context and matrices such as hair (Kaplan and Hunsberger, 2023). Alprazolam (Xanax), a high-potency BZD, is uniquely indicated for anxiety and panic disorders and non-FDA-labelled depression (George et al., 2025). Flunitrazepam (rohypnol), a benzodiazepine indicated for insomnia, is well documented in drug-facilitated crimes. In sexual assault cases, it is frequently present at low concentrations, often evading detection in victims (Dinis-Oliveira, 2017). These differences underscore the need for substance-specific forensics strategies.

Recently, there has been a surge in designer benzodiazepines (DBZDs). DBZDs are synthetic BZDs that are chemically modified and are capable of producing strong effects of amnesia and sedation compared to traditional BZDs. They can elevate the risk of fatal respiratory depression leading to death when used adjunctively with CNS depressants.

A few DBZDs used in crimes include clonazolam, diclazepam, etizolam, flualprazolam, flubromazepam, and phenazepam. Clonazolam found as a candy-like pill (half-life: 3.6 h) is more potent than alprazolam, while flualprazolam (half-life: 9.5–12 h) has increased sedation and overdose risk. DBZDs such as etizolam are undetectable by standard immunoassays, which require advanced.Liquid Chromatography–Tandem Mass Spectrometry (LC-MS/MS) for identification (Wilde et al., 2021). Their rapid metabolism and lack of clinical data pose unprecedented challenges in forensic investigations.Several designer analogues are not reliably detected by routine immunoassays; LC–MS/MS or adapted GC–MS protocols are needed to avoid under-ascertainment in DFC cases.

### Indications of benzodiazepines

Benzodiazepines are used to manage various anxiety disorders, including panic attacks and situational and generalized anxiety. Owing to their sedative properties, they are prescribed for the treatment of insomnia and for managing seizures. They are often administered to treat muscle spasms due to musculoskeletal or neurological conditions (Blanco et al., 2018). In contrast, benzodiazepines are contraindicated during pregnancy, particularly in the first trimester, as they are associated with neonatal hypotonia (Creeley and Denton, 2019). Heightened sensitivity and impaired clearance of the drug pose a greater risk of falls and delirium in older patients if prescribed benzodiazepines (By the 2023 American Geriatrics Society Beers Criteria^®^ Update Expert Panel, 2023).

### Effects of benzodiazepines on body systems and their adverse effects

BZDs have a detrimental effect, known as anterograde amnesia in the CNS, which alters memory formation and increases memory loss (Kaplan and Hunsberger, 2023). Patients with cardiovascular disorders have increased prevalence of depression and anxiety. BZDs reduce the autonomic hyperactivity of cardiac cells, which helps manage coronary heart disease by suppressing hypertension and myocardial ischemia (Balon et al., 2018).

As a result of CNS depression by benzodiazepines use, the muscle of the upper airway relaxes, leading to airway obstruction, which can worsen in patients with obstructive sleep apnea (Wang et al., 2019). Benzodiazepines can be used to treat anxiety disorders and panic attacks in patients with gastrointestinal symptoms. It reduces gastric secretion, which will ultimately help improve the signs and symptoms of peptic ulcers and cause relaxation of smooth muscle (Jembrek et al., 2017). As mentioned earlier, this drug causes neonatal sedation and withdrawal; hence, it is contraindicated in pregnant women (Creeley and Denton, 2019). Adverse events may occur if benzodiazepines are not well tolerated, with common manifestations including central nervous system effects such as sedation, dizziness, confusion, and memory impairment, including anterograde amnesia; respiratory effects such as hypoventilation and, in severe cases, respiratory arrest; cardiovascular effects including hypotension and bradycardia; gastrointestinal disturbances such as nausea and constipation; and musculoskeletal effects including weakness and ataxia (Kang et al., 2025).

### Drug-drug interactions

Patients are consistently warned against polypharmacy, particularly the elderly and those with chronic illnesses, because benzodiazepines exhibit synergistic depressant effects when combined with other central nervous system agents. These include antidepressants such as amitriptyline, anticonvulsants such as phenobarbital, sedative antihistamines such as diphenhydramine, barbiturates, and alcohol (Peppin et al., 2021). In contrast, theophylline can antagonize benzodiazepine-induced sedation, while drugs such as rifampin reduce benzodiazepine efficacy by inducing CYP3A4 metabolism (Brandt and Leong, 2017). Beyond therapeutic settings, polydrug use is highly relevant in DFCs, where benzodiazepines are rarely encountered in isolation. Alcohol potentiates benzodiazepine-induced anterograde amnesia, sedation, and psychomotor impairment, and combined use is consistently reported in case series of suspected DFSA, with blood ethanol frequently detected alongside therapeutic or even sub-therapeutic benzodiazepine concentrations (Pérez et al., 2023; Edinoff et al., 2021; Wilde et al., 2021). The interaction with opioids, especially synthetic analogues, is of even greater concern: concurrent use produces synergistic respiratory depression and is strongly associated with fatal outcomes in both epidemiological studies and forensic case reports (Edinoff et al., 2021; Jembrek et al., 2017; Brandt and Leong, 2017). In such toxicological investigations, plasma benzodiazepine concentrations that appear sub-toxic may nonetheless contribute to incapacitation or death when combined with ethanol or opioids (Edinoff et al., 2022; Wilde et al., 2021). Reviews of DBZDs similarly show frequent co-ingestion with alcohol and opioids in post-mortem casework, emphasizing the need for sensitive LC–MS/MS confirmation in specimens containing multiple drug classes (Pérez et al., 2023; Edinoff et al., 2022; Wilde et al., 2021).

### Dose-response relationship of benzodiazepines

BZDs, when administered at low doses, induce anti-anxiety effects and sedation and, to some extent, impair memory for as long as the half-life of the drug. Various types of BZDS and their properties have been described in Table 1. High doses of BZDs, or when administered with alcohol and barbiturates, can lead to severe adverse effects. A single dose of 0.5 mg3 Triazolam, a benzodiazepine, is associated with rebound insomnia, and repeated dosing can cause psychomotor disturbances and amnesia (Dujardin et al., 2020).

**TABLE 1:** Benzodiazepines: normal dose ranges, half-lives, principal indications, clinical toxicity features, and forensic considerations (Wilde et al., 2021; Bounds and Patel, 2025; Cloos et al., 2021; Van Amsterdam and Van den Brink, 2025; Pérez et al., 2023; Ntoupa et al., 2021; Borrelli et al., 2022; Al Bahri and Hamnett, 2023; Golts et al., 2025).

Drug	Half-life (h)	Normal dose (mg)	Principal indication(s)	Clinical toxicity features	Forensic considerations
Traditional benzodiazepines					
Diazepam	∼42	2–40	Anxiety, seizures, alcohol withdrawal	Ataxia, dysarthria, somnolence; coma with co-ingestants	Long half-life extends detection window; complicates timing of exposure
Alprazolam	6–20	0.5–4	Anxiety, panic disorder	Sedation, psychomotor impairment; aggression/disinhibition reported	High potency, frequently encountered in forensic casework
Lorazepam	∼12	1–10	Anxiety, status epilepticus	Marked CNS depression; respiratory compromise with depressants	Intermediate half-life; common in DFSA reports
Clonazepam	20–60	0.5–4	Seizure disorders, panic disorder	Somnolence, ataxia; paradoxical agitation uncommon	Detectable in hair due to long half-life
Midazolam	1.5–3	7.5–15	Insomnia, procedural sedation	Rapid sedation; respiratory arrest risk with co-ingestants	Very short half-life → narrow detection window in DFSA
Flunitrazepam	10–20	0.5–2	Insomnia (restricted use)	Profound amnesia; coma with co-ingestants	Repeatedly documented in DFSA and robbery cases
Temazepam	6–25	10–30	Insomnia	Next-day sedation, ataxia	Forensic significance when combined with alcohol
DBZDs					
Etizolam	3.4–7.1	0.5–3	Anxiety (prescribed in some countries)	Respiratory depression with co-ingestants	Often missed in immunoassays; requires LC–MS/MS
Flualprazolam	9.5–12	0.25–2	Designer use (no medical approval)	Potent sedation, amnesia	Designer analogue; detection requires MS-based methods
Clonazolam	∼3.6	0.25–2	Designer use (no medical approval)	Extreme potency; profound sedation and amnesia	Detection challenges without advanced testing
Phenazepam	15–60	0.5–2	Anxiety, epilepsy (former Soviet states)	Prolonged sedation; risk of accumulation	Very long half-life; detectable in hair samples

Other date-rape drugs, such as Ketamine and Gamma-hydroxybutyrate (GHB), differ from BZDs in their mechanism of action, whereas few clinical effects are similar to BZDs and are often misused (Morse et al., 2017, Nowak, 2015). GHB occurs naturally in the brain in small amounts but is misused in its synthetic form, and it acts on GHB receptors and GABAB, whereas ketamine is a synthetic anesthetic that acts by blocking NMDA receptors. All three drugs cause amnesia, and BZDs have the ability to cause anterograde amnesia. GHB overdose carries a markedly high risk of fatal respiratory depression, whereas ketamine use is associated with profound hallucinations. Due to its short half-life, GHB is not detected in standard toxicology drug screening, and ketamine requires specific testing to be detected (Germain et al., 2023). In contrast, BZDs are easily detected during drug screening and are legally used (Kang et al., 2025).

### BZDs in drug facilitated crimes

Crime rates have risen alongside the increased use of both DBZDs and traditional BZDs, although the causality remains under investigation. In 2020, the consumption of designer BZDs increased among frequent drug users as they faced shortages in prescription sedatives because of COVID-19 restrictions (Lethbridge, 2020; Zaami et al., 2022; Zaami et al., 2020). Recent studies have reported crime offenders to be mainly males within the average age group of 20–50 years, either with a history of mental illness or substance use disorder (Lethbridge, 2020; Albrecht et al., 2016). In one study, 79.3% of the participants were male, with ages ranging from 21 to 56 years. Among them, 89% were Australian and had a history of using three common benzodiazepines: diazepam (98.8%), temazepam (95.1%), and alprazolam (93.9%). The drugs were typically taken 12–24 h prior to incidents, indicating a short-acting window, and 98.8% of users consumed benzodiazepines in combination with other substances to enhance their effects (Albrecht et al., 2016). Among the traditional BZDs, alprazolam, commonly known as Xanax, is often abused in committing crimes owing to factors such as the addictive and short-acting properties of the drug, low lipophilicity, short half-life (Table 1), and a tendency to increase physical aggression (Ait-Daoud et al., 2018). Alprazolam was reported to have been used by 63% of people engaged in violent crimes and 58% of people involved in acquisitive crimes (Lethbridge, 2020). Another study on the serum concentrations of DBZDs in forensic cases reported 5700 criminal offences with BZDs detected in the blood of 575 cases. Among the reported drugs, diazepam was the most common DBZD detected in this study, and it was found that intake of concentrations of 0.010 mg/L or above was often followed by memory impairment in facilitating crimes (Heide et al., 2020). In a study that detected and quantified BZDs in spiked beverages, it was suggested that approximately 10–20 mg/L of diazepam dissolved in a beverage is used in drug-facilitated crimes (De Paula et al., 2018). Various methods are used to administer BZDs to sedate, incapacitate, and impair the memory of the victim in drug-facilitated crimes. They are most commonly administered orally by spiking drinks with BZD pills and less commonly administered via injections (Morgillo et al., 2023).

The elimination of benzodiazepines is governed by both Phase I oxidative metabolism and Phase II conjugation pathways, with marked differences across compounds. Phase I metabolism is primarily mediated by CYP3A4 and CYP2C19, generating active intermediates such as desmethyldiazepam that may prolong pharmacological activity, whereas Phase II glucuronidation facilitates clearance of hydroxylated derivatives including lorazepam, oxazepam, and temazepam (Griffin et al., 2013; George et al., 2025; Dinis-Oliveira, 2017). This metabolic diversity underlies clinically and forensically significant interindividual variability, influenced by genetic polymorphisms, hepatic impairment, age, sex, and comorbid conditions (Edinoff et al., 2021; Kaplan and Hunsberger, 2023; Dujardin et al., 2020). Such variability directly impacts toxicological interpretation, as two individuals may show divergent concentration–time profiles after identical doses. Acute exposure to short-acting benzodiazepines often produces narrow detection windows, with plasma levels falling below routine detection thresholds within hours. In contrast, chronic or repeated dosing promotes drug accumulation in adipose tissue and prolongs terminal elimination, extending the period of detectability in conventional matrices and enhancing incorporation into keratinized tissues such as hair and nails (Pérez et al., 2023; Fernández-López et al., 2024; Wilde et al., 2021). These pharmacokinetic and kinetic distinctions are critical in forensic practice, since the absence of a detectable analyte in blood or urine does not necessarily exclude prior exposure, particularly in delayed sampling or in individuals with rapid metabolic clearance.

### Pharmacogenetic and pharmacogenomic considerations

Evidence shows that CYP2C19 polymorphisms significantly affect diazepam metabolism: poor metabolizers may exhibit half-lives extended beyond 90 h, compared with the usual 30–50 h seen in extensive metabolizers (Griffin et al., 2013; Dinis-Oliveira, 2017). This prolongation results in higher plasma concentrations and longer detection windows, which can explain persistent positivity in urine or blood samples. Conversely, ultrarapid metabolizers may clear the drug within a markedly shorter timeframe, raising the possibility of false negatives in delayed sampling. Variability in CYP3A4 activity further influences the metabolism of alprazolam and triazolam, producing interindividual differences in both sedative effects and toxicological detectability (George et al., 2025). From a forensic standpoint, these pharmacogenetic differences can directly affect toxicological interpretation. A specimen collected 24–48 h after ingestion may remain positive in a poor metabolizer but negative in a rapid metabolizer, despite identical doses (Edinoff et al., 2021; Kaplan and Hunsberger, 2023; Dujardin et al., 2020).

### Forensic detection and analytical challenges

Forensic analysis of BZDs is becoming increasingly troublesome with a wider range of drugs, particularly with the advent of DBZDs (Zaami et al., 2022). Traditional analytical methods fail to identify new analogs; therefore, advanced methods are critical for their proper identification in toxicology, especially in cases of drug abuse or death (Catalan et al., 2021).

Benzodiazepines are lipophilic and well absorbed, but they are often present in biological matrices such as blood or urine at trace concentrations, particularly in postmortem samples or after a significant delay in sampling. Their extensive metabolic conversion produces multiple metabolites that may differ structurally and analytically from the parent compound, complicating detection and interpretation. Reliance on routine immunoassays can therefore result in false negatives or underreporting, particularly for short-acting benzodiazepines and novel designer analogues (Puzyrenko et al., 2022).

### Advanced analytical methods

Advanced analytical methods have improved sensitivity and reliability, yet interpretation still depends heavily on the matrix selected. Blood remains the reference specimen for correlating concentrations with acute impairment, but its detection window is limited. typically 6–24 h for short-acting agents such as triazolam and midazolam, while longer-acting compounds such as diazepam and nordazepam may remain detectable for several days depending on individual metabolic capacity (Griffin et al., 2013; Dinis-Oliveira, 2017). Urine is the most widely employed matrix due to its ease of collection and extended detection window, generally 2–5 days after single use and up to 1 week in chronic users, but results primarily demonstrate prior exposure and require enzymatic hydrolysis for reliable LC–MS/MS confirmation (Edinoff et al., 2021; Dujardin et al., 2020). Oral fluid provides a non-invasive alternative and approximates free plasma concentrations; however, instability and rapid analyte decline, alprazolam and lorazepam often falling below threshold within 12–18 h, limit its forensic reliability unless highly sensitive workflows are applied (Pérez et al., 2023; Edinoff et al., 2022; Wilde et al., 2021). In contrast, hair and nails permit retrospective detection over weeks to months, and segmental hair analysis can even distinguish single-dose ingestion from chronic intake (Pérez et al., 2023; Fernández-López et al., 2024; Wilde et al., 2021). Despite challenges related to external contamination, rigorous decontamination and confirmatory LC–MS/MS make these matrices invaluable in cases of delayed reporting, where blood and urine may yield false negatives.

While several biological specimens are available for benzodiazepine analysis, their utility differs markedly by detection window and forensic purpose. Blood and urine remain the primary matrices in acute investigations, whereas oral fluid provides a non-invasive but short-lived alternative. In cases with delayed reporting, hair and nails serve as valuable retrospective tools for documenting prior exposure. A comparative overview is presented in Table 2.

**TABLE 2:** Comparative properties of biological matrices in benzodiazepine detection.

Matrix	Detection window	Strengths	Limitations	Key references
Blood	6–24 h (short-acting BZDs); several days (long-acting)	Correlates with impairment; high specificity	Narrow window; invasive collection	Griffin et al. (2013), Dinis-Oliveira (2017)
Urine	2–5 days (single use); up to 1 week (chronic)	Easy to collect; inexpensive; broad detection	Indicates exposure, not impairment; requires hydrolysis	Edinoff et al. (2021), Dujardin et al. (2020)
Oral fluid	≤12–18 h for most BZDs	Non-invasive; reflects free plasma levels	Short window; analyte instability	Pérez et al. (2023), Edinoff et al. (2022), Wilde et al. (2021)
Hair	Weeks to months	Long-term detection; segmental timeline possible	Contamination/cosmetic bias	Pérez et al. (2023), Fernández-López et al. (2024), Wilde et al. (2021)
Nails	Months	Very long retrospective window	Low concentrations; less standardized	Pérez et al. (2023), Fernández-López et al. (2024), Wilde et al. (2021)

In suspected drug-facilitated sexual assault (DFSA), timely collection of blood and urine is essential, with confirmatory LC–MS/MS pursued whenever immunoassay screening is negative but clinical history strongly suggests benzodiazepine exposure. LC–MS/MS remains the gold standard for forensic toxicology owing to its high sensitivity and specificity. A single validated protocol has demonstrated the ability to detect 25 different benzodiazepines, including traditional and designer agents, in urine with nanogram-per-milliliter sensitivity, while simultaneously distinguishing among structurally similar compounds (Gqamana and Zhang, 2024; Whitehead et al., 2023).

Gas Chromatography-Mass Spectrometry (GC-MS) is a standard toxicological method but needs to be modified to detect thermally labile DBZDs that have been more recently introduced (Drug Enforcement Administration DEA, 2023). Altered GC-MS protocols have been successfully used to detect drugs such as bromazolam, flualprazolam, and etizolam in post-mortem blood. (Ballotari et al., 2025).

Ultraviolet–visible (UV-vis) spectroscopy provides enhanced sensitivity over conventional immunoassays for preliminary BZD detection capable of identifying trace concentrations in biological samples. Although useful for initial screening, the technique cannot reliably differentiate between structurally similar BZDs (such as alprazolam vs. etizolam). Current applications remain limited to specialized laboratories, where they serve as supplemental methods prior to confirmatory analysis by LC-MS/MS or GC-MS (Doctor and McCord, 2013).

Electrochemical nanosensor-electrochemical sensors utilizing glassy carbon electrodes (GCEs) modified with nanomaterials (gold nanourchins and Fe-Ni-doped reduced graphene oxide (Fe-Ni@rGO and AuNUs) demonstrate enhanced sensitivity for BZD detection. A validated nanosensor achieved a detection limit of 1 µg L−1 for alprazolam in blood serum, with minimal interference from structurally similar benzodiazepines, such as clonazepam (Sadrabadi et al., 2022). These sensors have the potential for rapid on-site screening, although their current applications remain limited.

LC-MS/MS remains the gold standard for BZD detection owing to its high sensitivity and specificity. However, challenges, such as unstable DBZDs and polydrug samples, have limitations. Modified GC-MS detects newer analogs, but struggles with heat-sensitive compounds. UV-vis spectroscopy offers improved screening but lacks specificity, while nanosensors show promise for onsite use but face adoption barriers. DBZDs are structurally very similar to traditional BZDs; thus, it is challenging to distinguish them using conventional screening equipment. Moreover, the unavailability of reference standards from commercial sources for new compounds prohibits their incorporation into toxicological profiles (Wu and Fu, 2023). It is also complicated when polydrug users consume several psychoactive drugs in a single sample, which may affect hide identification. All of these dynamics require continued development of approaches, international sharing of data among international toxicology laboratories, and investment in high-throughput holistic analytical platforms (Zhang et al., 2023).

### BZDs usage in the facilitation of robbery and theft cases

A glimpse at the numerous real-life cases documenting the dangerous side effects of benzodiazepines highlights the severity of the issue. In Brussels, mass robbery was carried out by intoxicating Asian travelers with cookies containing flunitrazepam. The side effects of sedation and amnesia due to the medicine led to theft of the victims’ belongings, as confirmed by the presence of the drug in victims’ systems via toxicological analyses. This case highlights the challenges of timely detection of benzodiazepines owing to their rapid metabolism (Ramadan et al., 2013). A similar case from India was documented, in which the victim was sedated with lorazepam and robbed. Analysis was performed using thin-layer chromatography (TLC) along with Fourier Transform Infrared Spectroscopy (FTIR) to confirm the presence of the drug, highlighting the importance of various forensic techniques in crime investigation (Ritu et al., 2018).

### BZDs usage in the facilitation of drug-facilitated sexual assault (DFSA) cases

The use of flunitrazepam in DFSA has been well documented. A case study reported on a female victim who was sexually assaulted by a male assailant after being offered a soft drink mixed with flunitrazepam. She experienced extensive retrograde amnesia, which led to difficulties in investigation. This showcased the complexities in legal proceedings due to memory impairment in victims (Ohshima, 2006). However, an interesting case report on a 56-year-old tourist who claimed to have been assaulted by five men after being given an alcoholic drink provided a solution to the former study. Seven months after the incident, the victim’s hair strands were collected and examined, revealing the presence of flunitrazepam and oxazepam. This case highlights the usefulness of hair analysis, which can play a crucial role in providing evidence, especially with a single exposure to such drugs even after a long period from the incident (Carfora et al., 2022). A criminal assault case of a 12-year-old girl who also tested positive for alprazolam in two hair segments and was assaulted after being sedated three to four times was documented (Kintz et al., 2005). A pilot study in Denmark reported 20 patients with DFSA, of which six were found to be positive for benzodiazepines via toxicological analyses (Birkler et al., 2012).

### BZDs usage in the facilitation of violence and homicide cases

Various cases have shown the relationship between benzodiazepine use and aggressive behavior in criminals. A 37-year-old man who demonstrated violent behavior following ingestion of benzodiazepines was reported in Malaysia. The assailant who suffered from schizophrenia attacked a woman with a sharp weapon, leading him to a 7-year jail sentence (Md Rosli and Singh, 2015). Similar results were demonstrated by a comparative study conducted among Houston Arrestees, in which benzodiazepine-positive detainees were more likely to be arrested for drug-related offences than non-users (Yacoubian et al., 2002). The murder of three people by a 23-year-old male student under the influence of flunitrazepam in Sweden has raised an important judicial dilemma. According to Swedish law, if a crime is committed under a severe mental disorder, the person will be sentenced to psychiatric care instead of prison. However, if the disorder is drug-induced, the individual can still be sentenced to prison. This raises a legal question in cases involving BZDs, which can mimic psychiatric conditions and also establishes the importance of proper investigations and fair sentencing (Dåderman et al., 2003).

**FIGURE 3:**
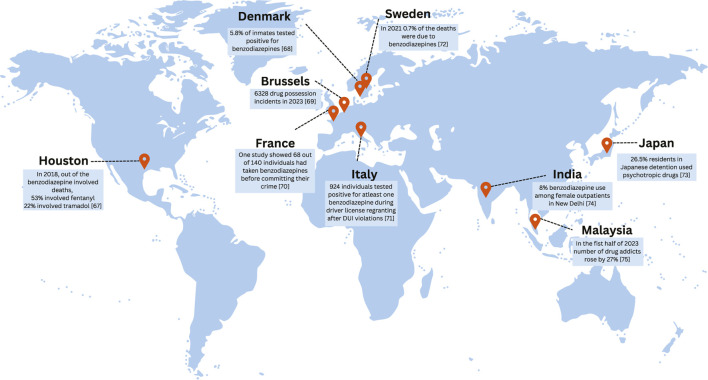
Illustration of the map highlighting regions with notable cases of BZD-facilitated crimes (Houston Texas Addiction Statistics, 2025; Brusselstimes.com, 2025; Masson, 2019; Kjær et al., 2024; Stefani et al., 2024; EUDA, 2024; Nishio, 2021; Deshpande and Nagpal, 1993; Tan, 2023).

Collectively, these cases span multiple regions, including Europe, Asia, and North America, demonstrating the global reach of BZD-facilitated crimes. Figure 3 depicts the regions with notable BZD-facilitated crime cases discussed in this section.

### Ethical and societal implications

Crimes involving BZDs, especially sexual assault, raise serious ethical and societal concerns. One major issue is victim-blaming, in which victims are held responsible for being intoxicated and their ability to consent is misunderstood. It is wrongly assumed that if someone is under the influence of drugs or alcohol, they are partially blamed. This discourages victims from reporting crime, thereby underreporting the problem (Pérez et al., 2023).

Stigma surrounding drug use and sexual assault is particularly strong in conservative societies (Pérez et al., 2023; Kennedy and O'Riordan, 2019). Public education is essential for changing these harmful attitudes. Campaigns should explain how BZDs affect memory and consent, and make it clear that being drugged removes a person’s ability to give informed consent (Kennedy and O'Riordan, 2019).

Therefore, prevention strategies need to be improved. People should be made aware of common methods used by drug victims, such as spiking drinks, and taught how to protect themselves, such as using drink-testing tools or avoiding unattended beverages. Public venues can also install better surveillance systems to help detect suspicious activities (Ohshima, 2006). Health professionals, especially those in emergency departments, must be trained to recognize the signs of drug-facilitated crimes. Many victims are not tested for BZDs because doctors are unaware of the issue or lack proper protocols (Kennedy and O'Riordan, 2019; Fløvig et al., 2010; Wu et al., 2024).

Therefore, there is an urgent need for legal strategies. Authorities should implement stricter regulations on BZD prescriptions, including mandatory Prescription Monitoring Programs (PMPs) that track high doses, risky combinations, and multiple prescribers in real time, particularly when enforced as mandatory rather than voluntary, to significantly reduce inappropriate prescribing. In addition, restricting pack sizes and high-dose formulations could further reduce excessive usage (Oldenhof et al., 2019).

Beyond prescription control, advanced forensic tools can help extend the window for drug identification. Law enforcement officers should receive training on the sensitive handling of suspected DFCs (Pérez et al., 2023; Wu et al., 2024). However, although these interventions are necessary, they remain insufficient. Systemic fragmentation across healthcare, law enforcement, and the public health sector accelerates the recurrent patterns of substance misuse, dependence disorders, and associated mortality. Only through an integrated multidisciplinary approach-incorporating policy reform, intersectoral collaboration, and standardized global protocols–can this cycle of misuse and dependence be effectively addressed.

## Conclusion

Designers and classic benzodiazepines are playing an increasingly important role in drug-facilitated crimes worldwide. Their potent sedative, anxiolytic, and amnesic effects in combination with the co-use of other psychoactive drugs constitute a dangerous scenario that can result in memory loss of victims, discredit testimony, and complicate criminal investigations. Forensic identification is difficult because of heavy metabolism, low quantities at the time of exposure, and continued synthesis of new designer analogs that may evade standard toxicological procedures.

This review summarized the neuropharmacological mechanisms that make BZDs a crucial tool for crime offenders. These mechanisms most commonly involve the potentiation of GABAergic signaling, resulting in anterograde amnesia and CNS depression. This review also highlights and describes the major analytical difficulties, emphasizing that although LC-MS/MS is the gold standard at the moment, newer methods such as electrochemical nano sensors hold promise for on-site applications in the future. This review also investigated the intricate legal and moral environments such as victim stigmatization and the use of memory impairment in testimony.

### Gaps, limitations, future directions, and innovations

Despite growing attention to BZD-facilitated crimes, major gaps remain in the detection and handling of these cases. A significant challenge is the short timeframe in which BZDs can be detected. Many of these drugs break down quickly in the body, especially short-acting drugs such as midazolam and alprazolam, making it difficult for forensic scientists to find evidence if testing is delayed. Standard tests using blood or urine often miss the drug if too much time has passed. Future research should focus on biometric sensors, portable nanotech-based testing devices, and onsite testing kits. These tools could allow victims or medical staff to test for BZDs shortly after exposure (Kintz et al., 2005).

Another limitation is the heavy reliance of the legal system on victim testimonies. Since BZDs can cause memory loss, victims often cannot recall what has happened. Legal reforms should emphasize forensic evidence, such as toxicology reports, rather than relying solely on personal memory (Birkler et al., 2012). Technology offers a promising solution to this problem. Artificial intelligence (AI) can improve drug detection by analyzing complex biological data and identifying drug traces more quickly and accurately by utilizing biometric sensors and portable nanotech-based testing devices. AI-powered security systems can also help accurately monitor drinking spiking. When combined with legal reforms and better public awareness, these innovations could greatly reduce the number of BZD-facilitated crimes and support victims in obtaining justice (Md Rosli and Singh, 2015).

Ultimately, a coordinated effort combining cutting-edge forensic science, more stringent prescription drug monitoring, public health education, and legal system reforms is required to address the complex threat of BZD-facilitated crimes. Communities can be better prepared to reduce the harm caused by these crimes and guarantee greater offender accountability by combining preventative measures, advanced detection technologies, and judicial awareness.

## References

[B1] Ait-Daoud N. Hamby A. S. Sharma S. Blevins D. (2018). A review of alprazolam use, misuse, and withdrawal. J. Addict. Med. 12 (1), 4–10. 10.1097/ADM.0000000000000350 28777203 PMC5846112

[B2] Al Bahri A. A. Hamnett H. J. (2023). Etizolam and its major metabolites: a short review. J. Anal. Toxicol. 47 (3), 216–226. 10.1093/jat/bkac096 36477341

[B3] Albrecht B. Staiger P. K. Best D. Hall K. Nielsen S. Lubman D. I. (2016). Benzodiazepine use of community-based violent offenders: a preliminary investigation. J. Subst. Use 22 (3), 295–303. 10.1080/14659891.2016.1195893

[B4] Ballotari M. Truver M. T. Baer D. C. Brogan S. C. Hoyer J. L. Crosby M. M. (2025). Identifying bromazolam, etizolam, and flualprazolam in blood using gas chromatography-mass spectrometry. J. Forensic Sci. 70 (3), 1105–1113. 10.1111/1556-4029.70003 39963811

[B5] Balon R. Rafanelli C. Sonino N. (2018). Benzodiazepines: a valuable tool in the management of cardiovascular conditions. Psychother. Psychosom. 87 (6), 327–330. 10.1159/000493015 30189429

[B6] Birkler R. I. Telving R. Ingemann-Hansen O. Charles A. V. Johannsen M. Andreasen M. F. (2012). Screening analysis for medicinal drugs and drugs of abuse in whole blood using ultra-performance liquid chromatography time-of-flight mass spectrometry (UPLC-TOF-MS)--toxicological findings in cases of alleged sexual assault. Forensic Sci. Int. 222 (1-3), 154–161. 10.1016/j.forsciint.2012.05.019 22770621

[B7] Blanco C. Han B. Jones C. M. Johnson K. Compton W. M. (2018). Prevalence and correlates of benzodiazepine use, misuse, and use disorders among adults in the United States. J. Clin. Psychiatry 79 (6), 18m12174. 10.4088/JCP.18m12174 30403446 PMC10309967

[B8] Borrelli E. P. Bratberg J. Hallowell B. D. Greaney M. L. Kogut S. J. (2022). Application of a diazepam milligram equivalency algorithm to assess benzodiazepine dose intensity in Rhode Island in 2018. J. Manag. Care Spec. Pharm. 28 (1), 58–68. 10.18553/jmcp.2022.28.1.58 34949119 PMC10373022

[B9] Bounds C. G. Patel P. (2025). “Benzodiazepines. 2024 Jan 30,” in StatPearls. Treasure Island, FL: StatPearls Publishing. PMID: 29261973. 29261973

[B10] Brandt J. Leong C. (2017). Benzodiazepines and Z-Drugs: an updated review of major adverse outcomes reported on in epidemiologic research. Drugs R. D. 17 (4), 493–507. 10.1007/s40268-017-0207-7 28865038 PMC5694420

[B11] Brusselstimes.com (2025). Brussels police see sharp increase in drug trafficking offences. Bruss. Times. Available online at: https://www.brusselstimes.com/brussels-2/1355008/brussels-police-see-sharp-increase-in-drug-trafficking-offences.

[B12] By the 2023 American Geriatrics Society Beers Criteria® Update Expert Panel (2023). American Geriatrics Society 2023 updated AGS Beers criteria® for potentially inappropriate medication use in older adults. J. Am. Geriatr. Soc. 71 (7), 2052–2081. 10.1111/jgs.18372 37139824 PMC12478568

[B13] Carfora A. Campobasso C. P. Cassandro P. Petrella R. Borriello R. (2022). Long-Term detection in hair of Zolpidem, oxazepam and flunitrazepam in a case of drug-facilitated sexual assault. J. Anal. Toxicol. 46 (1), e16–e20. 10.1093/jat/bkaa174 33180140

[B14] Catalani V. Botha M. Corkery J. M. Guirguis A. Vento A. Scherbaum N. (2021). The psychonauts' benzodiazepines; quantitative structure-activity relationship (QSAR) analysis and docking prediction of their biological activity. Pharm. (Basel). 14 (8), 720. 10.3390/ph14080720 34451817 PMC8398354

[B15] Cloos J. M. Lim Cow C. Y. S. Bocquet V. (2021). Benzodiazepine high-doses: the need for an accurate definition. Int. J. Methods Psychiatr. Res. 30 (4), e1888. 10.1002/mpr.1888 34331787 PMC8633930

[B16] Conway A. Rolley J. Sutherland J. R. (2016). Midazolam for sedation before procedures. Cochrane Database Syst. Rev. 2016 (5), CD009491. 10.1002/14651858.CD009491.pub2 27198122 PMC6517181

[B17] Creeley C. E. Denton L. K. (2019). Use of prescribed psychotropics during pregnancy: a systematic review of pregnancy, neonatal, and childhood outcomes. Brain Sci. 9 (9), 235. 10.3390/brainsci9090235 31540060 PMC6770670

[B18] Dåderman A. M. Strindlund H. Wiklund N. Fredriksen S. O. Lidberg L. (2003). The importance of a urine sample in persons intoxicated with flunitrazepam--legal issues in a forensic psychiatric case study of a serial murderer. Forensic Sci. Int. 137 (1), 21–27. 10.1016/s0379-0738(03)00273-1 14550609

[B19] De Paula C. Jurisch M. Piccin E. Augusti R. (2018). Recognizing drug-facilitated crimes: detection and quantification of benzodiazepines in beverages using fast liquid-liquid extraction with low temperature partitioning and paper spray mass spectrometry. Drug Test. Anal. 10 (2), 1348–1357. 10.1002/dta.2395 29663724

[B20] Deshpande S. N. Nagpal R. S. (1993). Benzodiazepine abuse among female outpatients in India. Addict. Behav. 18 (5), 595–596. 10.1016/0306-4603(93)90075-K 7906081

[B21] Dinis-Oliveira R. J. (2017). Metabolic profile of flunitrazepam: clinical and forensic toxicological aspects. Drug Metab. Lett. 11 (1), 14–20. 10.2174/1872312811666170407164216 28403803

[B22] Doctor E. L. McCord B. (2013). Comparison of aggregating agents for the surface-enhanced Raman analysis of benzodiazepines. Analyst 138 (20), 5926–5932. 10.1039/c3an00669g 23928656

[B23] Drug Enforcement Administration (DEA) (2023). National Forensic Laboratory Information System (NFLIS) annual report. Available online at: https://www.deadiversion.usdoj.gov.

[B24] Dujardin S. Pijpers A. Pevernagie D. (2020). Prescription drugs used in insomnia. Sleep. Med. Clin. 15 (2), 133–145. 10.1016/j.jsmc.2020.02.002 32386689

[B25] Edinoff A. N. Nix C. A. Hollier J. Sagrera C. E. Delacroix B. M. Abubakar T. (2021). Benzodiazepines: uses, dangers, and clinical considerations. Neurol. Int. 13 (4), 594–607. 10.3390/neurolint13040059 34842811 PMC8629021

[B26] Edinoff A. N. Nix C. A. Odisho A. S. Babin C. P. Derouen A. G. Lutfallah S. C. (2022). Novel designer benzodiazepines: comprehensive review of evolving clinical and adverse effects. Neurol. Int. 14 (3), 648–663. 10.3390/neurolint14030053 35997362 PMC9397074

[B27] EUDA (2024). Europa.eu. Age distribution of drug-induced deaths reported in the European Union, Norway and Türkiye in 2021 (percent). Available online at: https://www.euda.europa.eu/media-library/edr/2023/age-distribution-of-drug-induced-deaths-in-europe_en.

[B28] Fernández-López L. Rodríguez S. Cánovas-Cabanes A. Teruel-Fernández F. J. Almela P. Del Rincón J. H. (2024). Identification of benzodiazepine use based on dried blood stains analysis. Pharm. (Basel) 17 (6), 799. 10.3390/ph17060799 38931466 PMC11206677

[B29] Fløvig J. C. Vaaler A. E. Morken G. (2010). Effects of legal and illegal use of benzodiazepines at acute admission to a psychiatric acute department. BMC Res. Notes 3, 263. 10.1186/1756-0500-3-263 20958975 PMC2974733

[B30] García M. G. Pérez-Cárceles M. D. Osuna E. Legaz I. (2021). Drug-facilitated sexual assault and other crimes: a systematic review by countries. J. Forensic Leg. Med. 79, 102151. 10.1016/j.jflm.2021.102151 33773270

[B31] George T. T. Tripp J. (2025). in Alprazolam (Treasure Island (FL): StatPearls Publishing). Available online at: https://www.ncbi.nlm.nih.gov/books/NBK538165/. 30844192

[B32] Germain M. Desharnais B. Motard J. Doyon A. Bouchard C. Marcoux T. (2023). On-site drug detection coasters: an inadequate tool to screen for GHB and ketamine in beverages. Forensic Sci. Int. 352, 111817. 10.1016/j.forsciint.2023.111817 37741179

[B33] Golts V. A. Lebedev A. A. Blazhenko A. A. Lebedev V. A. Kazakov S. V. Bayramov A. A. (2025). Study of the effects of mammalian kisspeptin analogs and kisspeptin 10 in Danio rerio. Biol. Bull. Rev. 15, 201–208. 10.1134/S2079086424601327

[B34] Gqamana P. P. Zhang Y. V. (2024). High-Throughput quantitative LC-MS/MS analysis of benzodiazepines in human urine. Methods Mol. Biol. 2737, 103–111. 10.1007/978-1-0716-3541-4_10 38036814

[B35] Griffin C. E. Kaye A. M. Bueno F. R. Kaye A. D. (2013). Benzodiazepine pharmacology and central nervous system–mediated effects. Ochsner J. 13 (2), 214–223. Available online at: https://pmc.ncbi.nlm.nih.gov/articles/PMC3684331/. 23789008 PMC3684331

[B36] Heide G. Høiseth G. Middelkoop G. Øiestad Å. M. L. (2020). Blood concentrations of designer benzodiazepines: relation to impairment and findings in forensic cases. J. Anal. Toxicol. 44 (8), 905–914. 10.1093/jat/bkaa043 32369173 PMC7733327

[B37] Houston Texas Addiction Statistics (2025). Virtuerecoverycenter.com. Houston, Texas addiction statistics. Available online at: https://www.virtuerecoverycenter.com/houston-texas-addiction-statistics/August 16, 2025).

[B38] Iiorio M. T. Vogel F. D. Koniuszewski F. Scholze P. Rehman S. Simeone X. (2020). GABAA receptor ligands often interact with binding sites in the transmembrane domain and in the extracellular domain-can the promiscuity code be cracked? Int. J. Mol. Sci. 21 (1), 334. 10.3390/ijms21010334 31947863 PMC6982053

[B39] Jembrek M. J. Vlainic J. (2015). GABA receptors: pharmacological potential and pitfalls. Curr. Pharm. Des. 21 (34), 4943–4959. 10.2174/1381612821666150914121624 26365137

[B40] Jembrek M. J. Auteri M. Serio R. Vlainic J. (2017). GABAergic System in action: connection to gastrointestinal stress-related disorders. Curr. Pharm. Des. 23 (27), 4003–4011. 10.2174/1381612823666170209155753 28190395

[B41] Kang H. Dalager N. Mahan C. Ishii E. (2005). The role of sexual assault on the risk of PTSD among Gulf War veterans. Ann. Epidemiol. 15 (3), 191–195. 10.1016/j.annepidem.2004.05.009 15723763

[B42] Kang M. Galuska M. A. Ghassemzadeh S. (2025). in Benzodiazepine toxicity (Treasure Island (FL): StatPearls Publishing). Available online at: https://www.ncbi.nlm.nih.gov/books/NBK482238/. 29489152

[B43] Kaplan K. Hunsberger H. C. (2023). Benzodiazepine-induced anterograde amnesia: detrimental side effect to novel study tool. Front. Pharmacol. 14, 1257030. 10.3389/fphar.2023.1257030 37781704 PMC10536168

[B44] Kennedy K. M. O'Riordan J. (2019). Prescribing benzodiazepines in general practice. Br. J. Gen. Pract. 69 (680), 152–153. 10.3399/bjgp19X701753 30819759 PMC6400612

[B45] Kintz P. Villain M. Chèze M. Pépin G. (2005). Identification of alprazolam in hair in two cases of drug-facilitated incidents. Forensic Sci. Int. 153 (2-3), 222–226. 10.1016/j.forsciint.2004.10.025 16139113

[B46] Kjær T. L. Hindersson P. Bentzen J. R. Rasmussen H. H. Breindahl T. (2024). Drug use during incarceration: a comprehensive quality and prevalence study in three Danish prisons. Subst. Use Misuse 60, 155–167. 10.1080/10826084.2024.2421813 39482817

[B47] Lethbridge H. P. (2020). Benzodiazepine use and criminal activity: a case-crossover study dissertation. Hobart, Australia: University of Tasmania. Available online at: https://figshare.utas.edu.au/articles/thesis/Benzodiazepine_use_and_criminal_activity_a_case-crossover_study/23238167/1.

[B48] Masson E. (2019). Crime and benzodiazepine use, abuse and dependence. EM-Consulte. Available online at: https://www.em-consulte.com/article/1007715/crime-and-benzodiazepine-use-abuse-and-dependence-.

[B49] Md Rosli A. N. Singh S. (2015). Aggression following benzodiazepine ingestion in a forensic psychiatric patient: a case report. ASEAN J. Psychiatry 16 (2).

[B50] Mellen E. J. Kim D. Y. Edenbaum E. R. Cellini J. (2024). The psychosocial consequences of sexual violence stigma: a scoping review. Trauma Violence Abuse. Thousand Oaks, CA: SAGE Publications. 10.1177/15248380241279860 39377179

[B51] Morgillo A. Marovino E. Mazzarella M. Merandi S. Giordano L. Morgillo C. R. (2023). Old and “New Designer” benzodiazepines as crime facilitating drugs: a review of toxicological and analytical aspects. Qeios. 10.32388/3AZW0Q

[B52] Morohaku K. Phuong N. S. Selvaraj V. (2013). “Developmental expression of Translocator Protein/Peripheral benzodiazepine receptor in reproductive tissues,”PLoS ONE. Editor Yan W., 8. 10.1371/journal.pone.0074509 24040265 PMC3764105

[B53] Morse B. L. Chadha G. S. Felmlee M. A. Follman K. E. Morris M. E. (2017). Effect of chronic γ-hydroxybutyrate (GHB) administration on GHB toxicokinetics and GHB-induced respiratory depression. Am. J. Drug Alcohol Abuse 43 (6), 686–693. 10.1080/00952990.2017.1339055 28662343 PMC6103637

[B54] Nicholson M. W. Sweeney A. Pekle E. Alam S. Ali A. B. Duchen M. (2018). Diazepam-induced loss of inhibitory synapses mediated by PLCδ/Ca2+/calcineurin signalling downstream of GABAA receptors. Mol. Psychiatry 23 (9), 1851–1867. 10.1038/s41380-018-0100-y 29904150 PMC6232101

[B55] Nishio A. (2021). Psychotropic drug use rate among detention house residents and association with the category of the crimes in Japan. Neuropsychopharmacol. Rep. 41 (4), 464–470. 10.1002/npr2.12203 34432387 PMC8698679

[B56] Nowak A. (2015). Legal and criminological aspects of rape with date rape drug. Sci. J. Bielsko-Biala Sch. Finance Law 6 (4), 96–109. 10.5604/01.3001.0012.2982

[B57] Ntoupa P. S. A. Papoutsis I. I. Dona A. A. Spiliopoulou C. A. Athanaselis S. A. (2021). A fluorine turns a medicinal benzodiazepine into NPS: the case of flualprazolam. Forensic Toxicol. 39 (2), 368–376. 10.1007/s11419-020-00565-4

[B58] Ohshima T. (2006). A case of drug-facilitated sexual assault by the use of flunitrazepam. J. Clin. Forensic Med. 13 (1), 44–45. 10.1016/j.jcfm.2005.05.006 16087387

[B59] Oldenhof E. Anderson-Wurf J. Hall K. Staiger P. K. (2019). Beyond prescriptions monitoring programs: the importance of having the conversation about benzodiazepine use. J. Clin. Med. 8 (12), 2143. PMID: 31817181; PMCID: PMC6947397. 10.3390/jcm8122143 31817181 PMC6947397

[B60] Peppin J. F. Pergolizzi J. V. Raffa R. B. Wright S. L. (2021). The benzodiazepines crisis: the ramifications of an over-used drug class. Oxford University Press.

[B61] Pérez O. M. van Asten A. Kohler I. (2023). The evolution toward designer benzodiazepines in drug-facilitated sexual assault cases. J. Anal. Toxicol. 47 (1), 1–25. 10.1093/jat/bkac017 35294022 PMC9942444

[B62] Puzyrenko A. Wang D. Schneider R. Wallace G. Schreiber S. Brandt K. (2022). Urine drug screening in the era of designer benzodiazepines: Comparison of three Immunoassay platforms, LC-QTOF-MS and LC-MS-MS. J. Anal. Toxicol. 46 (7), 712–718. 10.1093/jat/bkab108 34557900

[B63] Ramadan A. S. Wenanu O. Cock A. D. Maes V. Lheureux P. Mols P. (2013). Chemical submission to commit robbery: a series of involuntary intoxications with flunitrazepam in Asian travellers in Brussels. J. Forensic Leg. Med. 20 (7), 918–921. 10.1016/j.jflm.2013.06.017 24112346

[B64] Ritu M. Komal S. Amit R. (2018). Forensic examination of benzodiazepines: a case Study. Open Acc. J. Toxicol. 3 (2), 555608. 10.19080/OAJT.2018.03.555608

[B65] Sadrabadi E. A. Khosravi F. Benvidi A. Shiralizadeh Dezfuli A. Khashayar P. Khashayar P. (2022). Alprazolam detection using an electrochemical nanobiosensor based on AuNUs/Fe-Ni@rGO nanocomposite. Biosens. (Basel). 12 (11), 945. 10.3390/bios12110945 36354454 PMC9687846

[B66] Stefani L. Mineo F. Romani L. Vernich F. Russo C. Marsella L. T. (2024). The prevalence of benzodiazepine use among Italian drivers in 15,988 cases of driving license regranting from 2015 to 2023: risks and implications for driving fitness. Separations 11 (6), 169. 10.3390/separations11060169

[B67] Tan T. (2023). Most abused drugs amphetamines, cannabis and opiates, says Home Ministry. The Star. Available online at: https://www.thestar.com.my/news/nation/2023/10/26/most-abused-drugs-amphetamines-cannabis-and-opiates-says-home-ministry.

[B68] Van Amsterdam J. Van den Brink W. (2025). Designer benzodiazepines: availability, motives, and fatalities. A systematic narrative review of human studies. Drug Alcohol Depend. 272, 112708. 10.1016/j.drugalcdep.2025.112708 40367553

[B69] Wang S. H. Chen W. S. Tang S. E. Lin H. C. Peng C. K. Chu H. T. (2019). Benzodiazepines associated with acute respiratory failure in patients with obstructive sleep apnea. Front. Pharmacol. 9, 1513. 10.3389/fphar.2018.01513 30666205 PMC6330300

[B70] Whitehead H. D. Hayes K. L. Swartz J. A. Lieberman M. (2023). Development and validation of a liquid chromatography tandem mass spectrometry method for the analysis of 53 benzodiazepines in illicit drug samples. Forensic Chem. 35, 100512. 10.1016/j.forc.2023.100512 37483533 PMC10358349

[B71] Wilde M. Auwärter V. Moosmann B. (2021). New psychoactive substances—Designer benzodiazepines. WIREs Forensic Sci. 3, e1416. 10.1002/wfs2.1416

[B72] Wu D. Fu L. (2023). Recent findings and advancements in the detection of designer benzodiazepines: a brief review. Arh. Hig. Rada Toksikol. 74 (4), 224–231. 10.2478/aiht-2023-74-3771 38146763 PMC10750316

[B73] Wu Y. Ma L. Li X. Yang J. Rao X. Hu Y. (2024). The role of artificial intelligence in drug screening, drug design, and clinical trials. Front. Pharmacol. 15, 1459954. 10.3389/fphar.2024.1459954 39679365 PMC11637864

[B74] Yacoubian G. S. Urbach B. J. Larsen K. L. Johnson R. J. Peters R. J. Jr (2002). Exploring benzodiazepine use among Houston arrestees. J. Psychoact. Drugs 34 (4), 393–399. 10.1080/02791072.2002.10399980 12562107

[B75] Zaami S. Marinelli E. Varì M. R. (2020). New trends of substance abuse during COVID-19 pandemic: an international perspective. Front. Psychiatry 11, 700. 10.3389/fpsyt.2020.00700 32765328 PMC7378810

[B76] Zaami S. Graziano S. Tittarelli R. Beck R. Marinelli E. (2022). BDZs, Designer BDZs and Z-drugs: pharmacology and Misuse Insights. Curr. Pharm. Des. 28 (15), 1221–1229. 10.2174/1381612827666210917145636 34533440

[B77] Zhang Y. X. Zhang Y. Bian Y. Liu Y. J. Ren A. Zhou Y. (2023). Benzodiazepines in complex biological matrices: recent updates on pretreatment and detection methods. J. Pharm. Anal. 13 (5), 442–462. 10.1016/j.jpha.2023.03.007 37305786 PMC10257149

